# Mitochondrial DNA Polymorphism A4917G Is Independently Associated with Age-Related Macular Degeneration

**DOI:** 10.1371/journal.pone.0002091

**Published:** 2008-05-07

**Authors:** Jeffrey A. Canter, Lana M. Olson, Kylee Spencer, Nathalie Schnetz-Boutaud, Brent Anderson, Michael A. Hauser, Silke Schmidt, Eric A. Postel, Anita Agarwal, Margaret A. Pericak-Vance, Paul Sternberg, Jonathan L. Haines

**Affiliations:** 1 Center for Human Genetics Research, Department of Molecular Physiology and Biophysics, Vanderbilt University Medical Center, Nashville, Tennessee, United States of America; 2 Department of Ophthalmology, Vanderbilt University Medical Center, Nashville, Tennessee, United States of America; 3 Center for Human Genetics, Duke University Medical Center, Durham, North Carolina, United States of America; 4 Duke Eye Center, Duke University Medical Center, Durham, North Carolina, United States of America; 5 The Institute for Human Genomics, Miller School of Medicine, University of Miami, Miami, Florida, United States of America; Peninsula Medical School, United Kingdom

## Abstract

The objective of this study was to determine if MTND2*LHON4917G (4917G), a specific non-synonymous polymorphism in the mitochondrial genome previously associated with neurodegenerative phenotypes, is associated with increased risk for age-related macular degeneration (AMD). A preliminary study of 393 individuals (293 cases and 100 controls) ascertained at Vanderbilt revealed an increased occurrence of 4917G in cases compared to controls (15.4% vs.9.0%, p = 0.11). Since there was a significant age difference between cases and controls in this initial analysis, we extended the study by selecting Caucasian pairs matched at the exact age at examination. From the 1547 individuals in the Vanderbilt/Duke AMD population association study (including 157 in the preliminary study), we were able to match 560 (280 cases and 280 unaffected) on exact age at examination. This study population was genotyped for 4917G plus specific AMD-associated nuclear genome polymorphisms in CFH, LOC387715 and ApoE. Following adjustment for the listed nuclear genome polymorphisms, 4917G independently predicts the presence of AMD (OR = 2.16, 95%CI 1.20–3.91, p = 0.01). In conclusion, a specific mitochondrial polymorphism previously implicated in other neurodegenerative phenotypes (4917G) appears to convey risk for AMD independent of recently discovered nuclear DNA polymorphisms.

## Introduction

Age-related macular degeneration (AMD) is the leading cause of irreversible severe vision loss in Caucasians over the age of 50.[1] Evidence from familial aggregation studies, twin studies, and segregation analysis all point to a significant role for genetic factors in the etiology of AMD.[2], [3] Among common genetic polymorphisms, the ApoE 2 allele appears to be associated with increased susceptibility to AMD.[4] Recent genetic research has focused primarily on the nuclear genome and resulted in significant new associations, such as those in the Complement Factor H gene (CFH) on Chromosome 1 and the HTRA1/LOC387715 gene complex on Chromosome 10, with the AMD phenotype.[5], [6] However, variation in the mitochondrial genome may also be important in the pathophysiology of age-related diseases. This small, circular genome consists of only 16,569 base pairs yet encodes for vitally important subunits in the mitochondrial electron transport chain as well as complete sets of tRNAs and rRNAs. [7] Mitochondria are cytoplasmic organelles that play a central role in cellular energy production, free radical production, and apoptosis. Each of these processes has been implicated in the pathogenesis of AMD. [8] Under normal physiologic conditions, electrons leak from the mitochondrial electron transport chain and reduce oxygen to superoxide anion, initiating a cascade of free radicals, called reactive oxygen species (ROS) that indiscriminately damage biological macromolecules.[9] These free radicals damage the mitochondria and thus can compromise the production of ATP and trigger apoptosis. Previous research has demonstrated that retinal pigment epithelium is especially susceptible to damage by ROS. [10] Antioxidants appear to ameliorate this effect.[10] Mitochondria are especially susceptible to damage by ROS because the mitochondrial genome has limited reparative capacity. [7] In addition, production of ROS, and hence susceptibility to AMD, may differ depending on genetic variation within an individual's mitochondrial genome.

Stable single nucleotide polymorphisms (SNPs) have emerged in the mitochondrial genome over the past 150,000 years. [11] Related combinations of these mtDNA polymorphisms define haplogroups whose distribution differs between continents and populations reflecting both human migration and acquired genetic variation. [11]

This genetic variation results in distinctive sets of human mitochondrial electron transport chains with different capacities for energy production, free radical generation and apoptosis. Variations in the mitochondrial genome have been associated with neurodegenerative disorders, including Parkinson disease, Alzheimer disease, Friedrich's ataxia, and amytrophic lateral sclerosis.[12] Mitochondrial haplogroup T has been associated with male infertility due to asthenozoospermia with decreased Complex I function.[13] In previous work we have associated mitochondrial haplogroup T and specifically A4917G (4917G), a non-synonymous mtDNA SNP closely linked to that haplogroup, with nucleoside reverse transcriptase inhibitor (NRTI)-associated peripheral neuropathy complicating therapy in HIV patients.[14], [15] We hypothesized that 4917G may be related to the neurodegenerative pathophysiology leading to the development of AMD.

## Materials and Methods

### Study Population Ascertainment

AMD patients and unrelated controls were ascertained at both Vanderbilt University Medical Center (VUMC) and Duke University Medical Center (DUMC). The study protocol had approval by the Institutional Review Boards at both institutions and followed the tenets of the Declaration of Helsinki. Informed consent was obtained from all participants.

The preliminary study group consisted of individuals ascertained exclusively at Vanderbilt (N = 393). Following the preliminary study, the study population was expanded to include all N = 1547 individuals in the combined Vanderbilt/Duke AMD study population. From this group, which included all individuals in the preliminary study, we matched on the exact age (in years) at the time of the examination in order to negate any confounding effect from the difference in age between cases and controls. We were able to successfully match on exact age for N = 560 individuals (280 cases and 280 controls). Of these 560 individuals, 157 were in the preliminary study (83 cases and 74 controls). The age-matched group was an attempt at the discovery of an association independent of other nuclear genetic confounders and free of confounding from differences in age at examination. In no way did this study represent a validation of the initial finding since individuals are in both groups. The preliminary data (N = 393) is presented simply to report what actually occurred.

### Clinical Assessment

A standard protocol was used throughout ascertainment at both locations. [16] A thorough medical history including ocular and dietary information was obtained at the time of enrollment. Additional information regarding specific risk factors for AMD was obtained by a health and activities survey. All patients and controls had complete ophthalmoscopic exams that included both a slit-lamp examination and biomicroscopy with a 90-diopter lens or fundus contact lens, and 20-diopter indirect ophthalmoscopy of the peripheral retina. Fundus photographs (color 35-mm, 3 fields minimum) were obtained from a minimum of three fields for all patients, including stereophotographs of the disk and macula.[15] Vitreoretinal disease specialists (EAP and AA) examined the fundus photographs of all participants and assigned grades based on a modification of the AREDS grading system. The overall grade for an individual was based on the grade of the most severely affected eye. For this study, cases were defined as individuals with grade 3 to grade 5 AMD. Controls had either Grade 1 or Grade 2.

### DNA Analysis

DNA was isolated from whole blood using PUREGENE (Gentra Systems Inc., Minneapolis, MN, USA). Genotyping was performed with the ABI PRISM 7900HT Sequence Detection System (Applied Biosystems Inc., Foster City, California, USA) using the 5′nuclease allelic discrimination *Taq*man assay. The mtDNA 4917G snp was detected by using a MGB Eclipse Probe because of a polymorphism at the position 4918 (Nanogen, Bothell, WA). The 4917 forward primer was CAACTGCCTGCTA*TGATGGAT and the 4917 reverse primer was GGCCTGCTTCTTCTCACATGACA (* represents a proprietary nucleotide analog). The FAM probe for the wild type 4917 allele was TTACGA*TT*A*GT*GN*GG and the TET probe for the 4917G allele was TTACGA*CTA*GT*GN*GG (with N and * representing proprietary nucleotide analogs.) Genotypic data were analyzed using ABI Sequence Detection System version 2.1 software and confirmed by visual inspection of the plots. The genotyping for the specific CFH, LOC387715, and ApoE polymorphisms were carried out using ABI assays that have been described elsewhere. [5], [6]

### Statistical Analysis

Mitochondrial 4917G genotype frequencies were compared between cases and controls using a χ^2^ test. The 4917G genotype frequencies were calculated as the proportion of cases or controls that carried A or G alleles. This polymorphism is generally homoplasmic (only one allele is present in a given individual). Tests for statistical significance were two-sided with an alpha level of 0.05. Multivariable logistic regression was used to assess the relationship between independent variables and the outcome. Effect size for the association is measured as odds ratio (OR) with 95% confidence intervals. Statistical Analysis Software version 9.1 (SAS Institute, Cary, NC, USA) was used for all statistical analyses.

## Results

The initial study population consisted of 393 individuals (293 cases and 100 controls) ascertained at Vanderbilt University Medical Center. The clinical characteristics of these individuals are presented in Table 1. All patients were Caucasian. The results of the genotyping of these individuals are also incorporated into Table 1. An increased occurrence of 4917G was observed in cases compared to controls (15.4% vs 9.0%, p = 0.11). The significant difference in age between cases and controls (77.5 years±7.5 vs. 67.8 years±8.3, p<0.001) led to the use of an age-matching strategy in the subsequent main study.

**Table 1:** Demographic, Clinical, and Genetic Characteristics of the Initial Study Population.

Variable	Cases	Controls	p-value
Number	N = 293	N = 100	
Age at Exam, mean±sd, years	77.5±7.5	67.8±8.3	<0.001
Race, % Caucasian	100	100	
Gender, % Female	63.8	58.0	0.300
rs1061170, CFH, %Allele C present	84.3	63.0	<0.0001
rs10490924 LOC387715, %Allele T present	63.1	50.0	0.021
APOE, % ApoE2 allele	16.4	16.0	0.929
mtDNA 4917, % G allele	15.4	9.0	0.115

Following this preliminary study, we selected 280 tightly age-matched Caucasian pairs from the existing large AMD study population ascertained at Vanderbilt and Duke University. The age at exam of both groups was 70.0 years±7.6. The univariate analysis of both clinical and genotype data in this population is presented in Table 2. This analysis revealed significant differences between cases and controls for 3 nuclear polymorphisms- CFH Y402H (rs1061170), LOC387715 (rs10490924), and ApoE, as well as the mitochondrial polymorphism 4917G. For nuclear polymorphisms, the reported Odds Ratios (OR) reflects carriage of the risk allele in either the homozygous or heterozygous state. In the case of the mitochondrial polymorphism 4917G, virtually all individuals (>99%) were homoplasmic for this polymorphism. The multivariate regression analysis of this study population is presented in Table 3. Following adjustment for gender and the aforementioned three nuclear polymorphisms in this age-matched Caucasian population, the mitochondrial 4917G polymorphism remained an independent predictor of AMD (OR = 2.16, 95%CI 1.20–3.91, p = 0.01).

**Table 2:** Univariate Analysis of MTND2*LHON4917G and other factors involved in the development of AMD in N = 280 Caucasian pairs matched on the exact Age at the Time of Examination.

Variable	Cases	Controls	p-value
Number	N = 280	N = 280	
Age at Exam, mean±sd, years	70.0±7.6	70.0±7.6	
Race, % Caucasian	100	100	
Gender, % Female	62.1	53.2	0.0328
rs1061170, CFH, %Allele C present	80.7	66.8	0.0002
rs10490924 LOC387715, %Allele T present	63.1	50.0	<0.0001
APOE, % ApoE2 allele	16.4	16.0	0.0547
mtDNA 4917, % G allele	15.4	9.0	0.0140

**Table 3:** Multivariate Analysis of MTND2*LHON4917G and other factors involved in the development of AMD in N = 280 Caucasian pairs matched on the exact Age at the Time of Examination.

Variable	Odds Ratio	95% CI	p-value
Gender, % Female	1.41	0.99–2.00	0.0567
rs1061170, CFH, %Allele C present	1.97	1.32–2.94	0.0009
rs10490924 LOC387715, Allele T present	1.95	1.38–2.77	0.0002
APOE, ApoE2 allele present	1.50	0.94–2.40	0.0807
mtDNA 4917, G allele present	2.16	1.20–3.91	0.0108

## Discussion

In this study, we observed that 4917G, a non-synonymous mitochondrial DNA polymorphism associated with mitochondrial haplogroup T, appears to confer an increased risk for AMD. This variation in the mitochondrial genome was an independent predictor of AMD following multivariate adjustment for well-known nuclear genetic factors. This observation should not be surprising, since mitochondria are vitally important in free radical production, apoptosis and cellular energy production. [17]–[19]

While this study provides epidemiologic evidence of relationship between mtDNA 4917G and susceptibility to developing AMD, the biological mechanism underlying this association remains to be elucidated. The nonconservative amino acid change resulting from the 4917G polymorphism in the ND2 gene may increase the rate of electron leakage from the mitochondrial electron transport chain. As a direct consequence, reactive oxygen species (ROS) production may increase and result in damage to many important biomolecules. [7], [8] Increased mitochondrial free radical production may therefore be an important shared feature in the pathogenesis of a variety of neurodegenerative phenotypes. [12] The mtDNA variations detected in late-onset diseases such as AMD and Parkinson disease may persist in the population because they do not have an effect sufficient to impair reproductive fitness.

Genetic factors other than 4917G are clearly important in the development and progression of AMD. Our analysis of other established genetic risk factors for AMD was consistent with the published literature. However, 4917G remained an independent predictor of AMD following adjustment for these known risk factors. It is possible that the 4917G mtDNA polymorphism is in linkage disequilibrium with more important causative polymorphisms. Of course, replication of these results in other populations of suitable sample size and power will provide even more convincing evidence for the role this mtDNA polymorphism plays in AMD. Epistatic interactions between individual loci within the mitochondrial genome as well as nuclear-mitochondrial gene interactions, for instance perhaps involving LOC387715, will be investigated further in larger studies. [20]

In summary, this study provides new evidence that variation in the mitochondrial genome contributes to susceptibility to AMD. The magnitude of the risk associated with the haplogroup T suggests that it may be an important new risk factor for the development of AMD. Future studies involving AMD will need to take into account the distribution of 4917G in their study populations. As we move forward with genome-wide association studies of AMD that span the 6,000,000,000 base pairs of the human diploid nuclear genome, it is worth noting again that humans have two genomes. [21], [22] Variations in the 16,569 base pairs of the mitochondrial genome should not be overlooked in the search to uncover new factors important in the development of AMD and other diseases.
